# Transient Midcavity Obstruction from Hyperdynamic Mid–Left Ventricular Segments during Recovery from Classical Takotsubo Syndrome

**DOI:** 10.1016/j.case.2026.05.003

**Published:** 2026-06-17

**Authors:** Miguel A. Woodham, Anthony DeMaria, Prerana Bhatia

**Affiliations:** Division of Cardiovascular Medicine, University of California, San Diego, San Diego, California

**Keywords:** Takotsubo syndrome, Wall motion abnormality, Recovery, Echocardiography, Cardiomyopathy

## Abstract

• TTS characteristically has LV apical ballooning.
• Recovery from this transient condition is highly variable.
• TTE features during recovery are not well described.
• Recovery phase of TTS can demonstrate hyperdynamic mid-LV segments.

## Graphical abstract

**Figure:**
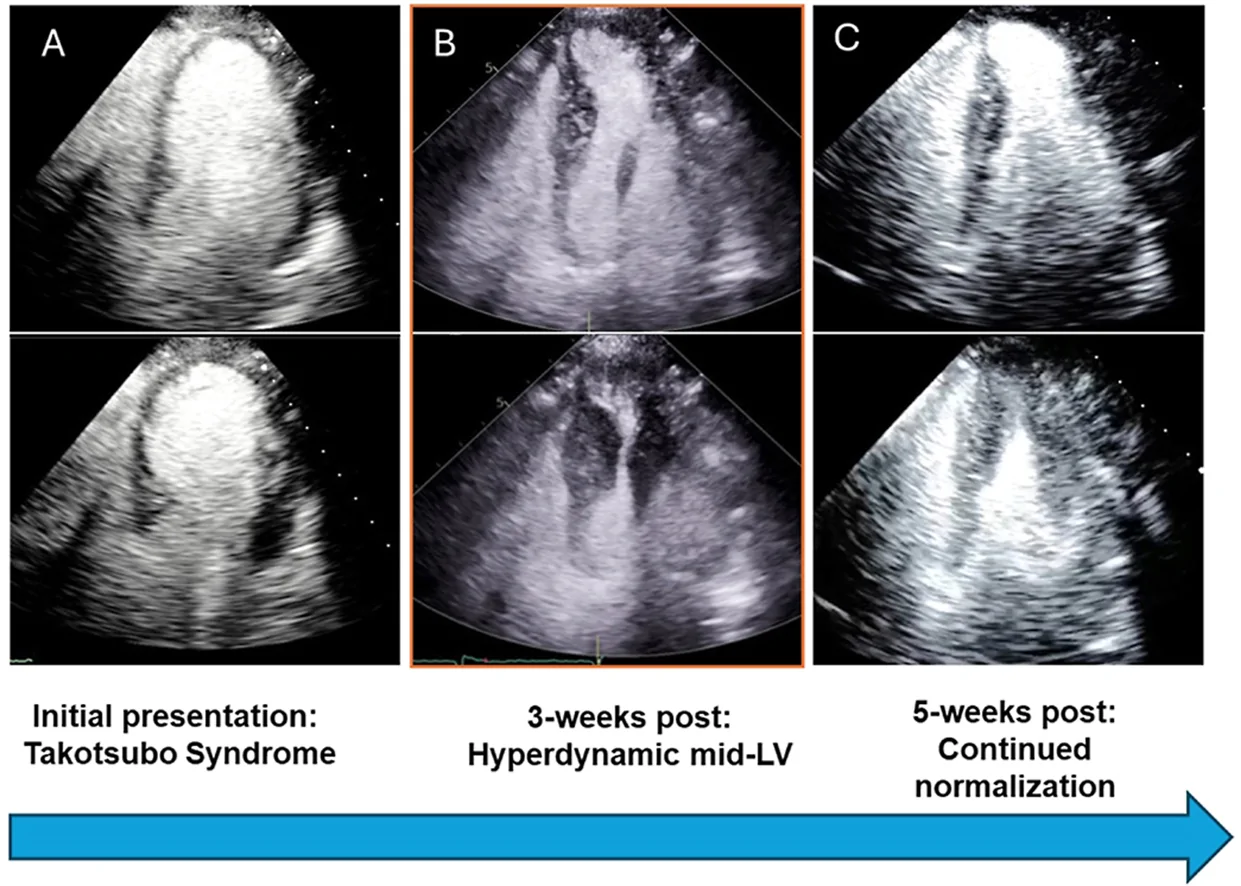

## Introduction

Takotsubo syndrome (TTS) is a transient cardiomyopathy characterized by reduced left ventricular (LV) systolic function with LV apical ballooning. Although recovery of myocardial stunning typically occurs within days to weeks, the morphologic evolution of contractile recovery during this period is incompletely characterized. Here we present a case of a female patient diagnosed with TTS with classic echocardiographic features of apical ballooning and mid-LV segment akinesis. The patient was noted to have unusually hyperdynamic mid-LV segments with midcavity obstruction 3 weeks after TTS diagnosis along with new orthostatic symptoms. Serial transthoracic echocardiography (TTE) showed complete recovery of the myocardium at a 6-month follow-up. Our case highlights contractile redistribution with transient dynamic midcavity obstruction during recovery from TTS as a potential mechanism of recurrent symptoms during the recovery phase.

## Case Presentation

An 84-year-old female patient presented to the emergency department with 4 days of shortness of breath at rest that worsened when lying flat and lower extremity swelling. The patient had been evaluated at an urgent care center 3 days prior due to cough and shortness of breath, diagnosed with community-acquired pneumonia, and prescribed doxycycline. The patient had a history of paroxysmal atrial fibrillation, sinus node dysfunction with a dual-chamber pacemaker in place, hypertension, and chronic kidney disease stage 3 (estimated glomerular filtration rate, 40 mL/min/1.73 m^2^). Vital signs on presentation were blood pressure, 139/93 mm Hg; pulse, 112 bpm; temperature, 99.5°F; respiratory rate, 35 breaths per minute; and SpO_2_, 98% on 2 L/min of oxygen via nasal cannula. On physical examination, there was jugular venous distension with an estimated pressure of 15 cm H_2_O, tachycardia and irregular rhythm, diffuse rhonchi on lung auscultation with increased work of breathing, and cool extremities. Electrocardiogram (ECG) showed atrial fibrillation with rapid ventricular response (heart rate, 131 bpm) with premature ventricular contractions and without ischemic changes (Figure 1A). Cardiac biomarkers were elevated with high-sensitivity troponin T Gen 5 assay at 229 ng/L (normal < 22) and N-terminal pro B-type natriuretic peptide at >18,000 pg/mL. Transthoracic echocardiography showed a severely reduced LV ejection fraction of 27% (measured via Simpson's biplane method using an ultrasound-enhancing agent) with apical and midsegment akinesis (Figure 2, Video 1). There was no LV outflow obstruction or systolic anterior motion of the mitral valve. Invasive coronary angiography showed no obstructive epicardial coronary artery disease (Figure 3). The patient was diagnosed with stress-induced cardiomyopathy, also known as TTS. No clear physical or emotional trigger was identified. The patient was medically treated for cardiogenic shock using dobutamine infusion with rapid stabilization and eventually transitioned to maintenance therapy. The patient was discharged home after 5 days on angiotensin receptor-neprilysin inhibitors, beta-blockers, mineralocorticoid receptor antagonists, and diuretics.

**Figure 1:**
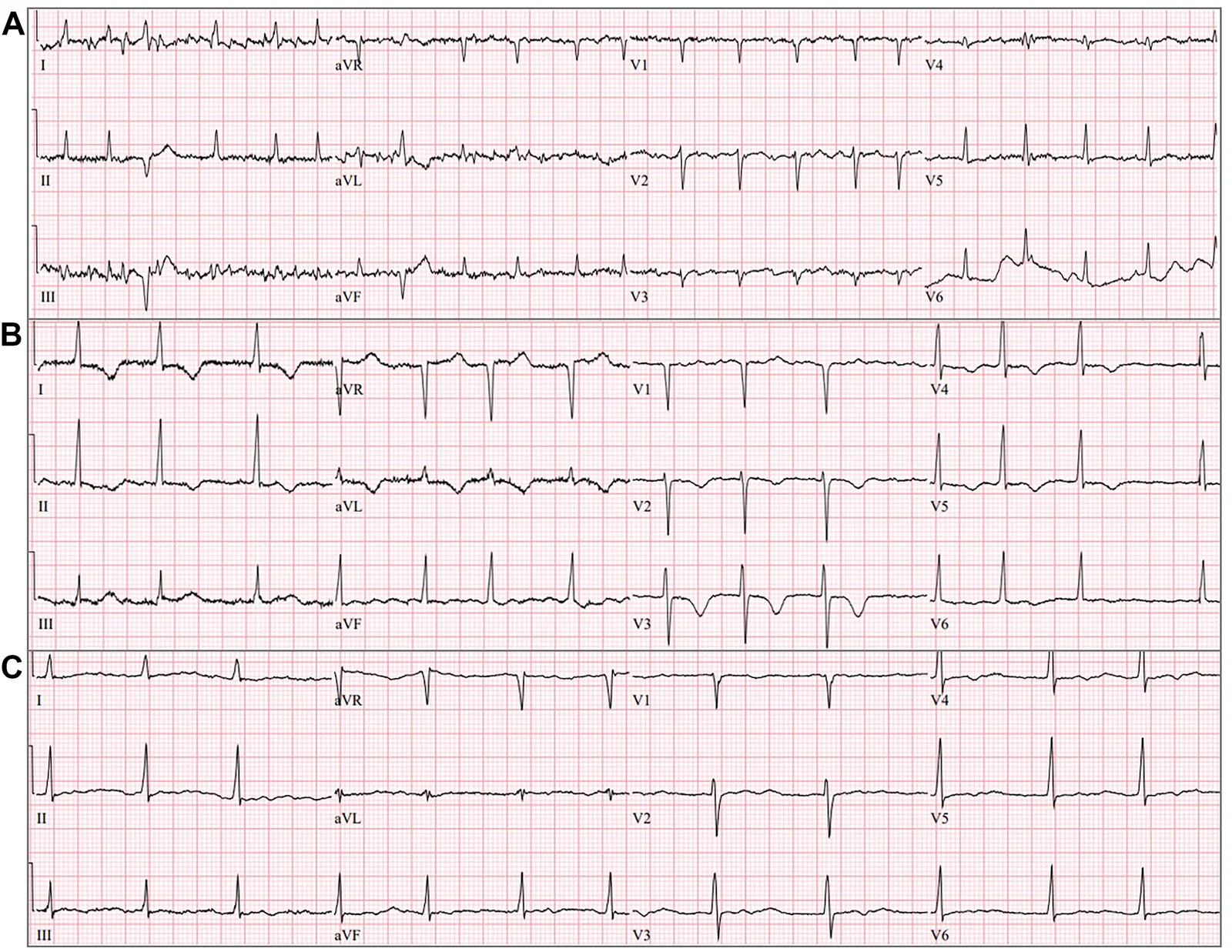
Serial 12-lead ECG tracings from index TTS presentation **(A)**, readmission 3 weeks later **(B)**, and follow-up 5 weeks after index event **(C)** demonstrate atrial fibrillation with rapid ventricular response, premature ventricular contractions, and nonspecific ST changes initially, new anterolateral T-wave inversions consistent with the evolving phase of TTS, and resolution of prior anterolateral T-wave inversions.

**Figure 2:**
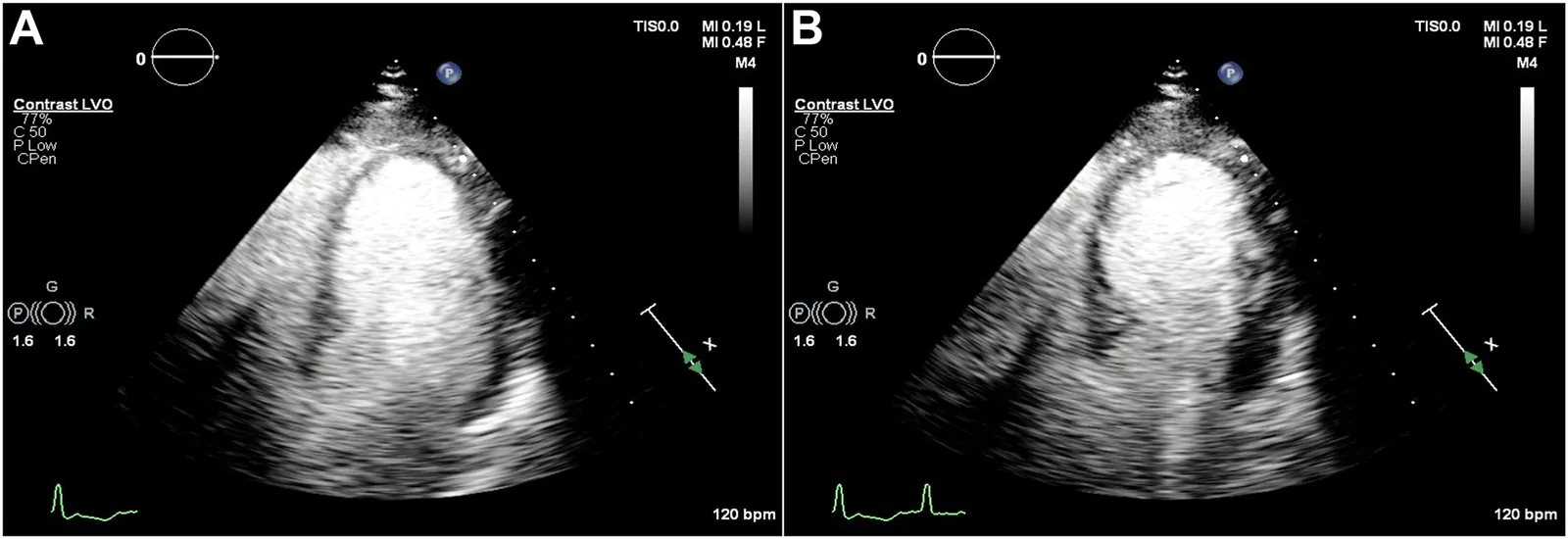
Two-dimensional TTE, apical 4-chamber diastolic **(A)** and systolic **(B)** views obtained at initial hospitalization after the administration of an ultrasound-enhancing agent, demonstrates severely reduced LV systolic function with apical and midsegment akinesis and ballooning consistent with classic TTS.

**Figure 3:**
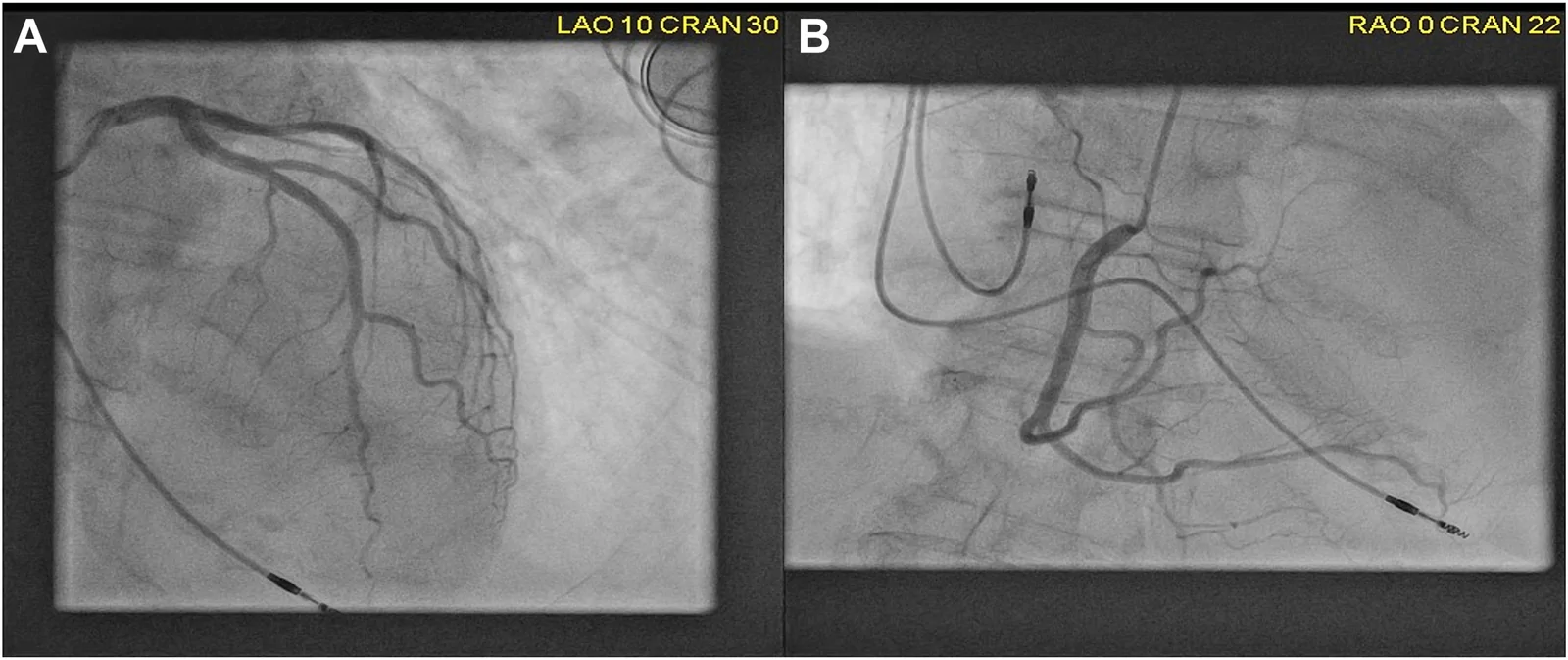
Invasive coronary angiography, left-anterior oblique cranial view with selective left coronary artery enhancement **(A)** and right-anterior oblique cranial view with selective right coronary artery enhancement **(B)**, demonstrates normal origin and course of native coronary arteries without obstructive coronary artery disease.

The patient presented to the emergency department again 3 weeks after the initial admission and diagnosis of TTS with progressive orthostatic lightheadedness. Symptoms significantly worsened 2 days before this second presentation, to the point where the patient was no longer able to get out of bed due to lightheadedness. The patient was noted to be hypotensive on arrival to the emergency department, with a blood pressure of 85/51 mm Hg that improved with an intravenous infusion of 500 mL of normal saline. After receiving additional intravenous fluids over several hours, totaling 1,250 mL of normal saline, the patient continued to have orthostatic symptoms with supine, seated, and standing blood pressures of 122/74, 122/72, and 97/64 mm Hg, respectively. Heart rate averaged 90 bpm without significant variation with position changes. Due to concern for possible LV outflow obstruction, TTE was repeated at this time and showed LV ejection fraction of 54% (measured via Simpson's biplane method using an ultrasound-enhancing agent) with new hyperdynamic motion in the mid-LV segments and apical hypokinesis (Figure 4, Video 2). Midcavity LV gradient was 60 mm Hg during supine condition at rest (Figure 5). There was no systolic anterior motion of the mitral valve leaflet or chordae tendineae. Inferior vena cava was 1.5 cm in diameter and collapsible. An ECG demonstrated T-wave inversions in the anterolateral leads (Figure 1B). High-sensitivity troponin T Gen 5 assay and N-terminal pro B-type natriuretic peptide were elevated but down-trending compared to the admission 3 weeks prior at 55 ng/L and 4,352 pg/mL, respectively. At this second presentation, several diagnostic considerations were entertained. Ischemia was considered less likely given the absence of anginal symptoms, down-trending cardiac biomarkers, and a recent invasive coronary angiography without obstructive coronary artery disease. Anterolateral T-wave inversions on ECG were felt to be consistent with the evolving course of TTS. Hypovolemia was also considered given the patient's orthostatic symptoms; however, this did not fully account for the observed regional wall motion abnormalities. Structural abnormalities, such as hypertrophic cardiomyopathy, were considered; however, there was no evidence of baseline LV hypertrophy on prior TTE, and the baseline ECG did not support underlying structural heart disease. The patient was treated for presumed orthostatic hypotension, and the angiotensin receptor-neprilysin inhibitors, mineralocorticoid receptor antagonists, and diuretics were stopped.

**Figure 4:**
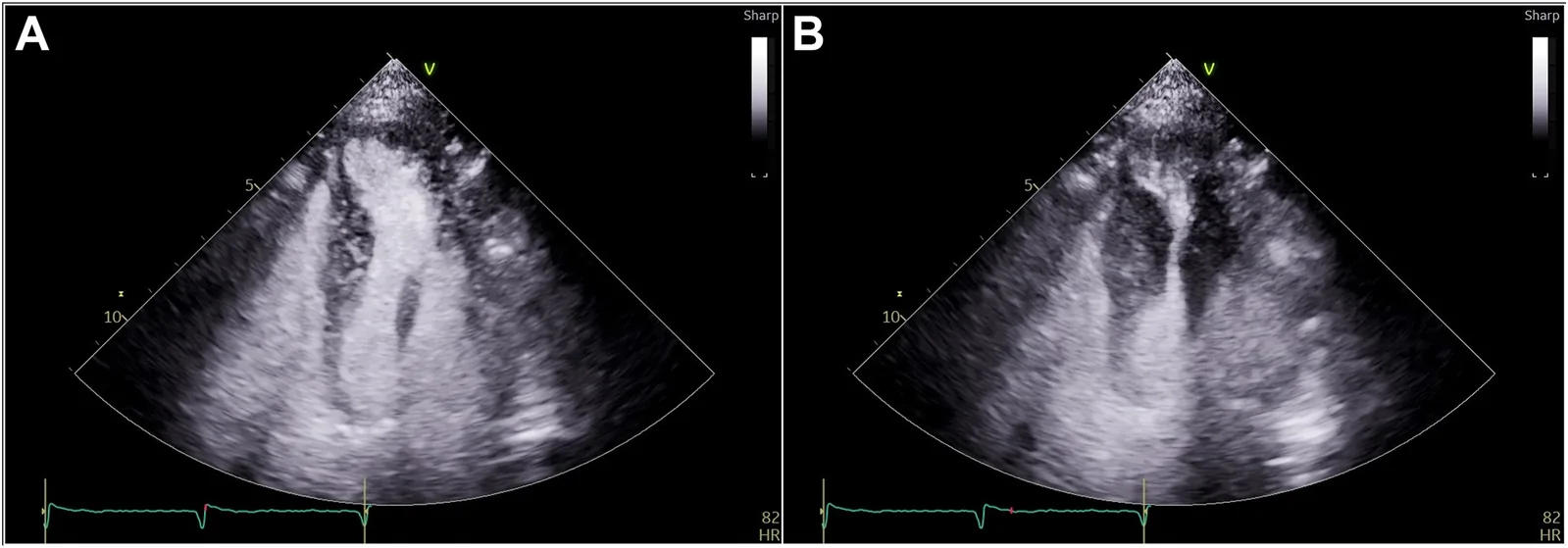
Two-dimensional TTE, apical 4-chamber diastolic **(A)** and systolic **(B)** views obtained during readmission 3 weeks after TTS diagnosis, after the administration of an ultrasound-enhancing agent, demonstrates a recovered normal LV systolic function with new systolic cavity obliteration and hyperdynamic motion in the mid-LV wall segments and persistent, but smaller, apical akinesis.

**Figure 5:**
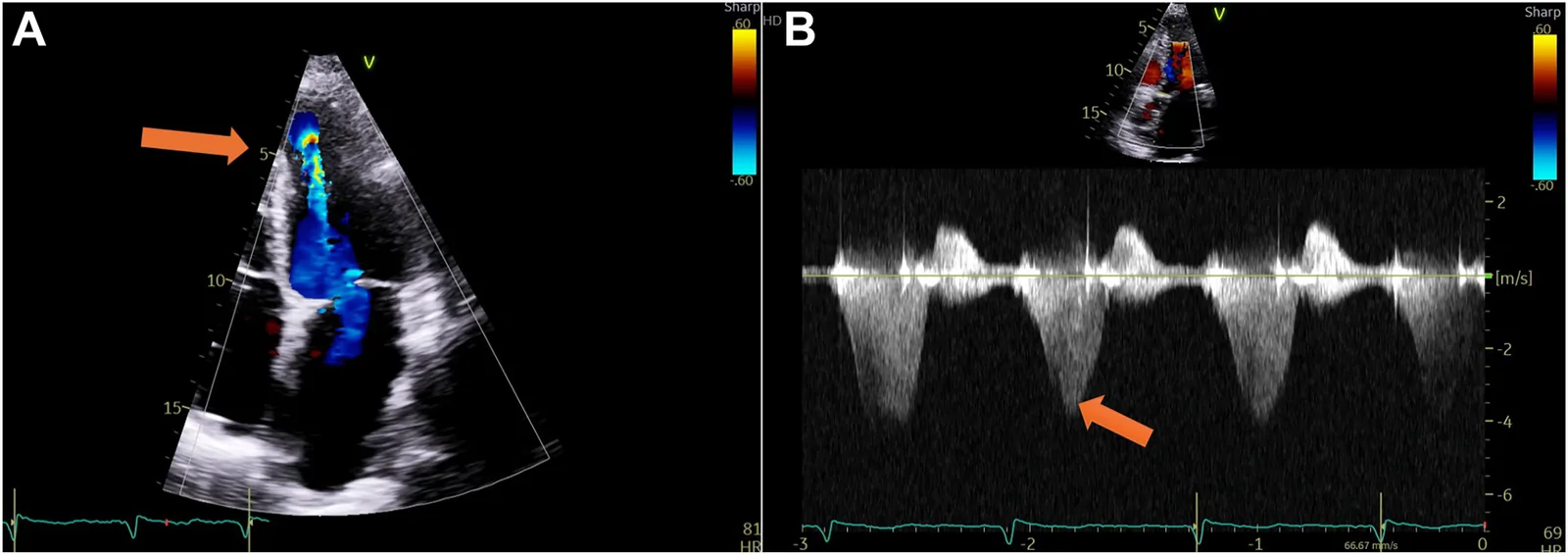
Two-dimensional TTE with color-flow Doppler, apical 4-chamber view **(A)** and continuous-wave Doppler spectral display **(B)** obtained during readmission 3 weeks after TTS diagnosis, demonstrates flow acceleration in the mid–left ventricle (*arrow* in **A**), which correlates with a midcavity gradient of 60 mm Hg (*arrow* in **B**).

A follow-up TTE was recommended due to the unusual hyperdynamic motion in the mid-LV segments and midcavity gradient. The patient had this follow-up TTE 2 weeks later (5 weeks after the initial presentation; Figure 6, Video 3), where apex ballooning had resolved and mid-LV segments were less hyperdynamic. Supine midcavity LV gradient decreased to 28 mm Hg (Figure 7). An ECG done around this 5-week period (Figure 1C) during clinic follow-up showed resolution of anterolateral T-wave inversions. Since the patient had no cardiac symptoms at this time, no further therapy adjustments were made. The patient then had a routine follow-up clinic visit and TTE at 6 months (Figure 8, Video 4) after the initial TTS diagnosis, which demonstrated normalization of the wall motion abnormalities, with no mid-LV obliteration or obstruction noted. Since this last TTE did not demonstrate underlying structural heart disease or outflow obstruction, routine surveillance imaging was not pursued. Instead, the patient was followed in a cardiology clinic at regular 6-month intervals with a plan for future imaging and testing to be symptom driven.

**Figure 6:**
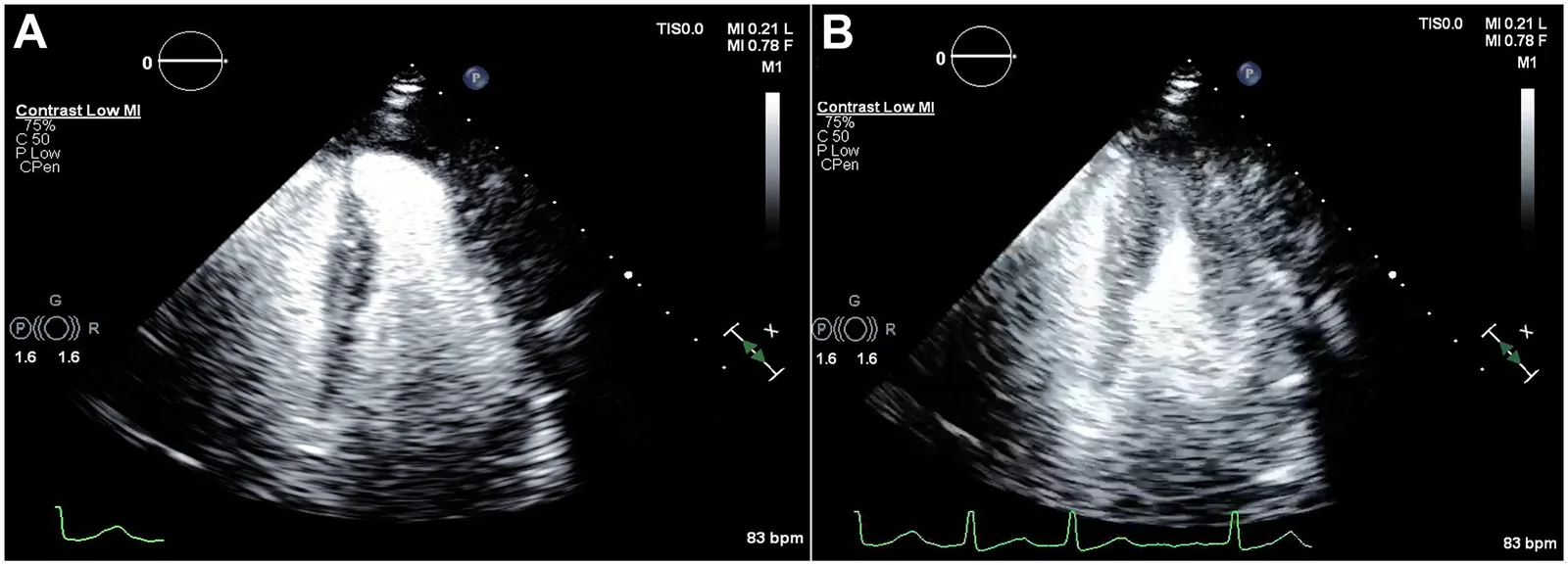
Two-dimensional TTE, apical 4-chamber diastolic **(A)** and systolic **(B)** views obtained 5 weeks after TTS diagnosis, after the administration of an ultrasound-enhancing agent, demonstrates normal global LV systolic function with near normalization of the LV apical function and morphology and resolution of the midcavity obliteration.

**Figure 7:**
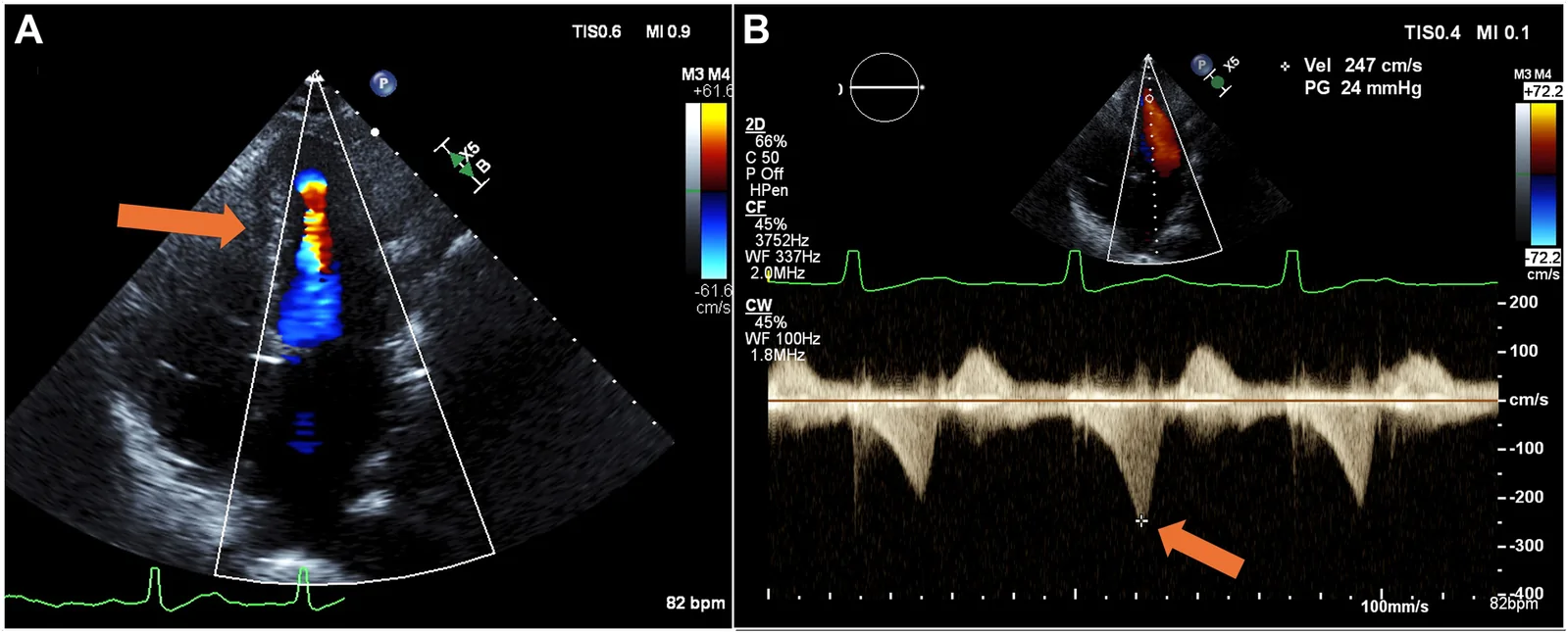
Two-dimensional TTE with color-flow Doppler, apical 4-chamber systolic view **(A)** and continuous-wave Doppler spectral display **(B)** obtained 5 weeks after TTS diagnosis, demonstrates flow acceleration in the mid–left ventricle (*arrow* in **A**), which correlates with a midcavity gradient of 28 mm Hg (*arrow* in **B**).

**Figure 8:**
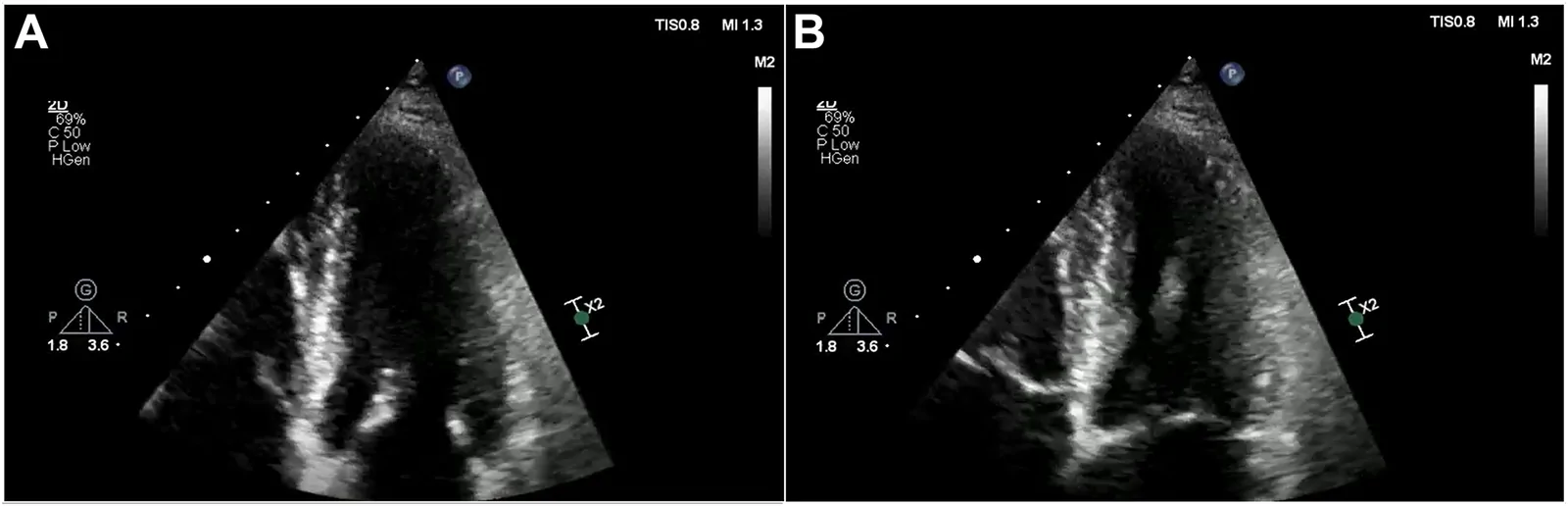
Two-dimensional TTE, apical 4-chamber diastolic **(A)** and systolic **(B)** obtained 6 months after TTS diagnosis, demonstrates normal global and regional LV systolic function.

## Discussion

Takotsubo syndrome is a transient stress-induced cardiomyopathy characterized by LV systolic dysfunction with apical ballooning in the absence of obstructive epicardial coronary artery disease.^1^^,^^2^ Takotsubo syndrome predominantly affects women and accounts for 2% to 3% of presentations with acute myocardial injury, often accompanied by symptoms and electrocardiographic findings that can masquerade as acute myocardial infarction.^2^, ^3^, ^4^ Although the pathophysiology of TTS remains incompletely understood, it is thought to involve heightened sympathetic activation and reduced vagal tone that places the myocardium at risk for the deleterious effects of elevated catecholamines.^5^ Recovery of myocardial stunning typically occurs over days to weeks, but the morphologic evolution of ventricular contractile recovery during this period is incompletely characterized.^6^ While ECG evolution and strain-based recovery patterns have been described, corresponding morphologic recovery patterns on conventional echocardiography remain poorly defined, likely reflecting substantial interpatient variability and heterogeneous mechanisms of myocardial injury, including catecholamine-mediated myocardial stunning, inflammation, and possible ischemic stunning.^5^^,^^7^, ^8^, ^9^, ^10^

We present a patient with classic mid and apical-LV akinesis consistent with TTS who transiently developed hyperdynamic mid-LV contraction with systolic midcavity obliteration and intraventricular gradient formation 3 weeks after the initial diagnosis. The combination of persistent apical hypokinesis and hypercontractile midventricular segments resulted in a dynamic intraventricular gradient occurring in the absence of hypertrophic cardiomyopathy or systolic anterior motion of the mitral valve leaflet or chordae tendineae. Serial TTEs demonstrated progressive resolution of the midcavity gradient and complete normalization of the LV morphology by 6 months.

This case illustrates a recovery-phase contractile redistribution after TTS. The hyperdynamic LV segments observed during the recovery phase may represent regional heterogeneity in β-adrenergic signaling, catecholamine sensitivity, and evolving microvascular dysfunction, resulting in temporally heterogeneous myocardial recovery.^5^^,^^10^^,^^11^ Recovered segments may transiently demonstrate hyperdynamic contraction while adjacent regions remain hypokinetic, predisposing to intraventricular gradient formation. The prevalence and reproducibility of this recovery-phase pattern remain unknown, highlighting an important knowledge gap.

Prior reports of midcavity obstruction in TTS have largely described an acute-phase phenomenon associated with altered ventricular geometry, dynamic intraventricular gradients, increased apical wall stress, and relative subendocardial ischemia. This physiology may be further accentuated in the setting of hypovolemia.^12^ In contrast, this case demonstrates midcavity obstruction emerging during the recovery phase in the absence of septal hypertrophy or systolic anterior motion of the mitral apparatus. While hypovolemia may have contributed to gradient severity, the overall pattern suggests contractile redistribution during recovery, rather than primary gradient-mediated ischemia.

This clinical presentation may be understood within the framework of a recovery-phase contractile redistribution phenotype, in which regional hypercontractility and persistent hypokinesis create dynamic intraventricular gradients that contribute to symptomatic hypotension and dizziness. Relative hypovolemia related to diuretic and vasodilator therapy may have amplified gradient severity; however, because these therapies were discontinued after the gradient was identified, their role in the initial development of this physiology remains uncertain. Resolution likely reflects a combination of evolving loading conditions and intrinsic myocardial recovery. Recognition of this physiology has important implications for management. Therapies that increase contractility or reduce preload, including positive inotropes, vasodilators, and aggressive diuresis, may exacerbate intraventricular gradients. In contrast, cautious volume optimization and beta-blockade may be beneficial. In hypotensive patients, short-acting vasopressors that increase afterload without augmenting contractility (e.g., phenylephrine) may be considered.^13^

An important clinical implication of this case is the need to interpret echocardiographic findings within the temporal context. Single-time-point imaging may not capture the evolving ventricular phenotype in TTS. In the absence of prior imaging, recovery-phase hyperdynamic midventricular segments with intraventricular gradients may be misinterpreted as hypertrophic cardiomyopathy variants or new ischemia, potentially leading to misdiagnosis or inappropriate management. This diagnostic challenge has been highlighted by prior reports of TTS occurring in patients with preexisting hypertrophic obstructive cardiomyopathy.^14^ In contrast, the current case demonstrates complete resolution of obstruction, absence of baseline or follow-up hypertrophy, and normalization of electrocardiographic findings, which argue strongly against a fixed structural substrate. Although recurrent TTS with different variants has been reported, the short interval between presentations, continuous biomarker down-trending, and progressive normalization on serial imaging favor a single recovery-phase process rather than recurrent TTS.^15^

We recognize several limitations. Cardiovascular magnetic resonance imaging was not performed, which may have provided additional tissue characterization. Limited endocardial definition on nonenhanced TTE precluded assessment of LV global longitudinal strain, which could have further highlighted or quantified the trends in regional wall motion abnormalities.

## Conclusion

Morphological LV changes during recovery from TTS remain incompletely characterized. This case demonstrates that transient mid-LV hypercontractility with dynamic midcavity obstruction can occur during recovery from TTS and represents a form of contractile redistribution rather than new structural pathology. Recognition of this recovery-phase physiology is clinically important, as it may explain recurrent symptoms such as dizziness or hypotension, inform therapy adjustment, and help prevent misdiagnosis in the absence of serial imaging.

## Consent Statement

The authors declare that since this was a non-interventional, retrospective, observational study utilizing de-identified data, informed consent was not required from the patient under an IRB exemption status.

## Ethics Statement

The authors declare that the work described has been carried out in accordance with The Code of Ethics of the World Medical Association (Declaration of Helsinki) for experiments involving humans.

## Funding

The authors declare that this report did not receive any specific grant from funding agencies in the public, commercial, or not-for-profit sectors.

## Disclosure Statement

The authors reported no actual or potential conflicts of interest relative to this document.

## Supplementary Data

Video 1 Two-dimensional TTE, apical 4-chamber **(A)** and apical 2-chamber **(B)** views obtained at initial hospitalization after the administration of an ultrasound-enhancing agent, demonstrates severely reduced LV systolic function with apical and midsegment akinesis and ballooning consistent with classic TTS.

Video 2 Two-dimensional TTE, apical 4-chamber **(A)** and apical 2-chamber **(B)** views, obtained during readmission 3 weeks after TTS diagnosis, after the administration of an ultrasound-enhancing agent, demonstrates normal LV systolic function with new systolic cavity obliteration and hyperdynamic motion in the mid-LV wall segments and persistent, but smaller, apical akinesis.

Video 3 Two-dimensional TTE, apical 4-chamber **(A)** and apical 2-chamber **(B)** views obtained 5 weeks after TTS diagnosis after the administration of an ultrasound-enhancing agent, demonstrates normal global LV systolic function with near normalization of LV apical function and morphology and resolution of the midcavity obliteration.

Video 4 Two-dimensional TTE, apical 4-chamber **(A)** and apical 2-chamber **(B)** views obtained 6 months after TTS diagnosis, demonstrates normal global and regional LV systolic function.
